# Health Literacy among Visitors of District Polyclinics in Almaty, Kazakhstan

**Published:** 2017-08

**Authors:** Gaukhar KAYUPOVA, Botagoz TURDALIYEVA, Kazbek TULEBAYEV, Tuyen Van DUONG, Peter Wushou CHANG, Diana ZAGULOVA

**Affiliations:** 1. Dept. of Healthcare Policy and Management, SD Asfendiyarov Kazakh National Medical University, Almaty, Kazakhstan; 2. School of Public Health, Taipei Medical University, Taipei, Taiwan; 3. National Taipei Hospital, Ministry of Health and Welfare, Taipei, Taiwan; 4. Baltic International Academy, Riga, Latvia

**Keywords:** Health literacy, Health behavior, Determinants of health literacy, Kazakhstan

## Abstract

**Background:**

This study aimed to evaluate health literacy levels of patients in Almaty City, Kazakhstan and to identify socio-demographics and socio-economic factors related to their health literacy.

**Methods:**

An international survey instrument HLS-EU-Q developed by the European Health Literacy Consortium was used in a cross-sectional study with 1000 citizens in the Almaty City at the age of 18 and over who visited the out-patient departments in the polyclinics between Feb and Oct 2014.

**Results:**

There were 552 women and 446 men completed the survey, with mean ages as (41.8 ± 13.9) and (44.7 ± 15.2) yr old respectively, and women were significantly younger than men (*P*<0.001). Their general health literacy was (34.0 ± 8.6) for men and (33.49 ± 9.4) for women, without significant difference. In them, 15.5% or 30.0% were with inadequate or problematic health literacy. Multivariate linear regression analysis showed that higher general health literacy was positively and significantly associated with high self-assessed social status (B=3.86, *P*<0.001), ability to pay for medications (B=3.42, *P*<0.001), low frequency of watching health related TV programs (B=2.37, *P*<0.001), moderate community involvement (B=2.23, *P*=0.03).

**Conclusion:**

Specific demographic and socio-economic determinants related to health literacy were identified the first time in Kazakhstan. This would facilitate programs to improve health outcomes in Kazakhstan.

## Introduction

Individuals’ characteristics and behaviors are the determinants of health among the social, economic, and physical environment (1). Among risk factors that are responsible for the differences between countries in the burdens of diseases tobacco, alcohol, high blood pressure, high cholesterol, overweight, low fruit and vegetable intake and physical inactivity (2). Thus, behavior changes towards healthier lifestyle and elimination of risky behavior are essential to reach better health outcomes of the population. Behavior change towards healthier lifestyle is recognized as the shared responsibility for individuals’ health, which depends significantly on their health literacy.

“Health literacy is linked to literacy and entails people’s knowledge, motivation, and competencies to access, understand, appraise, and apply health information in order to make judgments and take decisions in everyday life concerning healthcare, disease prevention and health promotion to maintain or improve quality of life during the life course” (3). European Health Literacy Consortium (HLS-EU Consortium) developed a conceptual model that included main aspects of health literacy, capturing the dimensions of health literacy within health care, disease prevention, and health promotion settings (4). On the other hands, health literacy is being increasingly researched in the world (5–9). Associations of health literacy and health outcomes had been observed in different age groups in different countries. In general, worse health behaviors were observed in children with low literacy, while parents and caregivers with low literacy had less health knowledge, compared with parents with higher literacy (10–12). Adolescents with low health literacy were less likely to perceive good health status and less likely to exhibit health-promoting behaviors (13). The elderly with higher health literacy scores were significantly less likely to have risky behaviors and more likely to undergo health examinations regularly, report good self-rated health, and to access sufficient health information from multiple sources (5, 14). Individuals with higher levels of HL had better health and welfare, more actively participated in economic prosperity, and more contributed to the society (4). Thus, health literacy is important both at the individual and at the society levels.

At the same time, higher proportions with limited health literacy were observed among subgroups with financial deprivation, low social status, low education, or old age indicating the presence of a social gradient (15). Therefore, increasing levels of health literacy will promote development of more capacity of each individual in health behaviors, realization of shared responsibility for one’s health, individual’s development towards improving quality of life. At the society level, increasing health literacy will contribute to the development of equity and sustainability of changes in public health (16).

Relatively little is known about health literacy of population in Kazakhstan and it has not been measured with internationally validated instrument so far. The present study aimed to evaluate health literacy of the residents in Almaty city who visited outpatient clinics during the survey.

The Health literacy research team of S Asfendiyarov Kazakh National Medical University (KazNMU) within the frames of the International project initiated by Asian Health Literacy Association (AHLA) conducted a survey using the conceptual-based comprehensive questionnaire HLS-EU-Q to evaluate different aspects of health literacy.

## Materials and Methods

### Study design and sampling

This cross-sectional study was conducted in Feb-Oct, 2014, inviting one each polyclinic in each district of all the 7 districts in the Almaty city. The trained interviewers recruited the participants over 18 yr old by randomly (mechanical sampling) enrollment of every second visitor out of those who visited the outpatient departments of these district polyclinics. Proportional sampling by different age groups were not conducted, while the subjects were invited in the survey when they visited the polyclinic, and their ages were proportional to the visitors to this polyclinic during the period, and further classified into different groups with 10 yr increment for further analysis. Among those who was invited to participate were not only individuals with some health problem but also those who came for the health check-up or for other non-treatment goals. The polyclinic - a primary healthcare organization- was chosen, as these were the key primary healthcare setting in Almaty.

### Questionnaires

The instrument HLS-EU-Q47 was developed by the European Health Literacy Consortium (3, 16), and extended by Asian Health Literacy Association (AHLA) to 106 questions. This version of HLS EU-Q was validated on a population level in Taiwan and elsewhere (5, 6). The questionnaire was translated professionally into Kazakh and Russian using translation-back translation method.

### Data collection

A target total 1000 individuals agreed to participate in the survey. The participants were asked to fill in the questionnaires anonymously with the assistance (if needed) of the trained interviewers-physicians and nurses in the polyclinics and the questionnaires were collected on site. Overall, 998 questionnaires were included in the analysis.

### Data analysis

The HL indices were calculated as the General health literacy (GHL), healthcare health literacy (HC-HL), disease prevention health literacy (DPHL), and health promotion health literacy (HPHL) (3, 16). Calculation of the indices was performed by the formula:

(formula 1) Index= (mean−1)*(50/3)

Where *Index* is the specific index calculated, *Mean* is the mean of all participating items for each individual, *1* is the minimal possible value of the mean (leading to a minimum value of the index of 0), *3* is the range of the mean, and *50* is the chosen maximum value of the new metric. An index value is obtained where 0 represents the lowest possible HL and 50 the highest possible HL (17).

Four levels of health literacy were defined as inadequate (0–25 points), problematic (>25–33 points), sufficient (>33–42 points), excellent (>42–50 points). Inadequate and problematic HL yielded limited HL(33points or less), sufficient and excellent HL yield satisfactory HL(> 33–50 points) (16).

To investigate the level of health literacy of the participants, descriptive analyses were performed. To identify associations between health literacy and various factors, bivariate and multivariate linear regression models were used.

(formula 2) $$Y=B_{0}+B_{1*}X_{1}+B_{2*}X_{2}+\ldots +B_{k*}X_{k}$$

While, Y is variation of dependent variables (Health literacy or health outcomes), B_0_ = intercept, B_1__→ k_ = Change of Y when X_1__→ k_ change 1 unit or between reference group and testing group.

### Ethical approval

The study was approved by Local Ethical Committee, S Asfendiyarov Kazakh National Medical university, Registration No 55.

## Results

### Characteristics of the participants

The mean age of women and men were 41.8 ±13.9 and 44.7 ±15.2 yr old, respectively. The personal and socio-demographic characteristics were shown in Table 1.

**Table 1:** Socio-demographics and characteristics of participants in Kazakhstan

**Characteristics**	**Men (N =446)**		**Women (N =552)**		**Overall (N =998)**	
	**n**	**Percentage**	**n**	**Percentage**	**n**	**Percentage**
Socio-demographics						
Age (yr)						
18–25	40	9.13	43	8.16	83	8.60
26–35	88	20.09	142	26.94	230	23.83
36–45	92	21.00	126	23.91	218	22.59
46–55	80	18.26	117	22.20	197	20.41
56–65	113	25.80	81	15.37	194	20.10
> 65	25	5.71	18	3.42	43	4.46
Educational attainment						
Junior high school and below	76	18.54	60	11.63	136	14.69
Senior high school	93	22.68	85	16.47	178	19.22
University and above	241	58.78	371	71.90	612	66.09
Ability to pay for medication						
Very difficult	8	2.03	10	2.12	18	2.08
Fairly difficult	27	6.84	24	5.10	51	5.89
Fairly easy	282	71.39	309	65.61	591	68.24
Very easy	78	19.75	128	27.18	206	23.79
Self-perceived social status						
Low	180	50.28	205	51.64	385	50.99
Middle	109	30.45	99	24.94	208	27.55
High	69	19.27	93	23.43	162	21.46
Personal behaviors						
Watch health-related TV						
Never	80	20.89	67	13.81	147	16.94
Rarely	143	37.34	175	36.08	318	36.64
Sometimes & Often	160	41.78	243	50.10	403	46.43
Community involvement						
Never	189	50.40	228	52.90	417	51.74
Rarely	53	14.13	61	14.15	114	14.14
Sometimes	32	8.53	44	10.21	76	9.43
Often	101	26.93	98	22.74	199	24.69
Health status						
Self-reported health status						
Very poor & Poor	39	9.44	35	7.14	74	8.19
Satisfactory	145	35.11	159	32.45	304	33.67
Good	148	35.84	182	37.14	330	36.54
Very good	81	19.61	114	23.27	195	21.59
Long-term illness						
None	213	52.59	277	57.83	490	55.43
One or more	192	47.41	202	42.17	394	44.57
Physical limitation related to health problem						
Not at all	193	50.66	249	54.01	442	52.49
Limited	188	49.34	212	45.99	400	47.51
Health behaviors						
Smoking status						
Current smoker	45	12.23	48	10.48	93	11.26
Former smoker	110	29.89	107	23.36	217	26.27
Non-smoker	213	57.88	303	66.16	516	62.47
Frequencies of visiting doctors						
None	32	8.65	56	12.81	88	10.90
1–2 times	54	14.59	65	14.87	119	14.75
3–5 times	186	50.27	222	50.80	408	50.56
6 times and more	98	26.49	94	21.51	192	23.79
Accompany to see doctors						
None	129	36.03	157	38.39	286	37.29
Sometimes	201	56.15	200	48.90	401	52.28
Often	28	7.82	52	12.71	80	10.43

55.3% of them were female participants, 66.1% had University education and above. 68.24% replied “it was fairly easy” and 23.8% “very easy” to pay for medications. 49.0% self-assessed social status as middle and high, 36.6% rarely and 16.9% never watched health related TV programs. 51.7% reported none community involvement, 62.5% non-smokers, 58.1% self-assessed health “very good and excellent”, 55.4% none long-term illness, 23.8% with 6 or more visits to doctors in the last 12 months, and 50.6% with 3 to 5 visits.

All Pearson’s coefficients for the total sample were reasonably high (minimum was 0.73 for the correlation between HP-HL and HC-HL).

General health literacy (GHL) was 34.0 ± 8.6 for men and 33.5 ± 9.4 for women, the HC-HL 34.4 ± 9.2 for men and 33.5 ± 10.5 for women, DPHL 34.2 ± 9.5 for men and 33.1 ± 10.5 for women, and HP-HL 33.5 ± 9.5 for men and 33.1 ± 9.9 for women. There was no statistically significant difference in health literacy level between men and women in the general HL and three sub-domains.

### Distribution of different levels of health literacy

Out of all the study population, 15.5% had inadequate GHL, 30.0% had problematic GHL, 36.1% had sufficient GHL, and 18.5% had excellent GHL.

For HC-HL, 16.1% were inadequate, 31.1% problematic, 33.1% sufficient, and 19.7% excellent. In DP-HL, 16.2% were inadequate, 27.7% problematic, 33.7% sufficient, and 22.5% excellent. In HP-HL, 17.5% were inadequate, 26.4% problematic, 34.3% sufficient, and 21.8% excellent. In them, higher GHL was positively and significantly associated with high self-assessed social status (*B* =3.86, *P* <0.001), ability to pay for medications (*B* =3.42, *P* <0.001), rarely watching health related TV programs, as compared to the respondents who never watched health related TV programs (*B* =2.37, *P* <0.001), moderate community involvement (*B* =2.23, *P* =0.03).

For men, higher GHL were positively and significantly associated with ability to pay for medications (*B* =4.9, *P* <0.001), rare community involvement (*B* =4.32, *P* <0.001), high self-assessed social status (*B* =3.68, *P* <0.001), rarely watching health related TV programs, as compared to the respondents who never watched health related TV programs (*B* =2.32, *P*=0.02), and age (*B* =0.66, *P*=0.02). For women, higher GHL was positively associated with ability to pay for medications (*B* =6.07, *P*=0.01), high (*B* =4.1, *P*<0.001) and middle (*B* =2.52, *P*=0.03) self-assessed social status, moderate community involvement (*B* =3.23, *P* =0.03), and rarely watching health related TV as compared to the respondents who never watched health related TV programs (*B* =2.77, *P*=0.04; Table 2).

**Table 2:** General health literacy associated with the socio-demographics and personal behaviors, by multivariate linear regression analysis ^a^

**Predictors**	**Men (n =446)**			**Women (n =552)**			**Overall (n =998)**		
	**B (95% CI)**	**β**	***P* value**	**B (95% CI)**	**β**	***P* value**	**B (95% CI)**	**β**	***P* value**
Socio-demographics									
Age with 10 yr increment	0.66 (0.09, 1.22)	0.12	0.02	−0.13 (−0.86, 0.61)	−0.02	0.74	0.41 (−0.05,0.87)	0.06	0.08
Marital status									
Not married (reference)									
Married, divorced, widow	1.43 (−0.51, 3.37)	0.08	0.15	−0.92 (−3.26, 1.42)	−0.04	0.44	−0.01 (−1.51, 1.5)	0.00	0.99
Educational attainment									
Junior high school and below (reference)									
Senior high school	0.84 (−1.55, 3.22)	0.04	0.49	1.91 (−1.36, 5.17)	0.07	0.25	1.39 (−0.56, 3.34)	0.06	0.16
University and above	1.43 (−0.54, 3.4)	0.09	0.15	1.32 (−1.32, 3.96)	0.06	0.33	1.08 (−0.52, 2.68)	0.06	0.19
Ability to pay for medication									
Fairly difficult & Very difficult (reference)									
Fairly easy	3.4 (0.91, 5.89)	0.19	0.01	4.26 (0.08, 8.44)	0.20	0.05	3.42 (1.21, 5.63)	0.18	<0.001
Very easy	4.9 (1.93, 7.87)	0.24	<0.001	6.07 (1.7, 10.44)	0.28	0.01	5 (2.57, 7.44)	0.24	<0.001
Self-perceived social status									
Low (reference)									
Middle	−0.06 (−1.81, 1.69)	0.00	0.95	2.52 (0.28, 4.76)	0.11	0.03	1.31 (−0.09, 2.72)	0.06	0.07
High	3.68 (1.77, 5.59)	0.19	<0.001	4.1 (1.94, 6.27)	0.18	<0.001	3.86 (2.41, 5.31)	0.18	<0.001
Personal behaviors									
Watch health-related TV									
Never (reference)									
Rarely	2.32 (0.34, 4.3)	0.14	0.02	2.77 (0.15, 5.39)	0.14	0.04	2.37 (0.75, 3.99)	0.13	<0.001
Sometimes & Often	1.27 (−0.67, 3.21)	0.08	0.20	1.01 (−1.58, 3.6)	0.05	0.44	0.88 (−0.7, 2.46)	0.05	0.28
Community involvement									
Never (reference)									
Rarely	4.32 (1.77, 6.87)	0.16	<0.001	−1.13 (−3.8, 1.54)	−0.04	0.41	0.96 (−0.88, 2.81)	0.03	0.31
Monthly	0.96 (−1.8, 3.72)	0.03	0.49	3.23 (0.35, 6.11)	0.10	0.03	2.23 (0.22, 4.23)	0.07	0.03
Often (everyday, several times a week)	0.88 (−0.87, 2.63)	0.05	0.32	−1.8 (−3.92, 0.32)	−0.08	0.10	−0.45 (−1.82, 0.92)	−0.02	0.52

Abbreviation: CI, confidence interval.

a B, non-standardized coefficient; β, standardized coefficient.

With multivariate linear regression analyses, the GHL as a predictor and its associated factors as dependent variables, GHL was positively and significantly associated with self-perceived health status (*B* =0.21, *P*<0.001), doing exercises (*B* =0.12, *P*<0.01), but negatively with smoking (*B* = −0.13, *P*<0.001), physical limitation related to health problem (*B* =−0.12, *P*<0.001), long-term illnesses (*B* =−0.08, *P* <0.01), and frequency of visiting doctors (*B* =−0.03, *P*<0.01; Table 3). In men, GHL was negatively associated with long-term illness(*B* =−0.12, *P*<0.01), smoking (*B* =−0.12, *P*<0.01), physical limitation related to health status (*B* =−0.11, *P*<0.05), but positively doing physical exercises (*B* =0.11, *P*<0.01). In women, their GHL was positively associated with self-perceived health status (*B* =0.34, *P*<0.001), but negatively with smoking (*B* =−0.16, *P*<0.001), physical limitation related to health problem (*B* =−0.13, *P*<0.0), having somebody to accompany them to visit a doctor(*B* =−0.07, *P* <0.05), frequency of visiting doctors (*B* =−0.05, *P*<0.001; Table 3).

**Table 3:** General health literacy (as a predictor) and its associated factors (as dependent variables) via multivariate linear regression analyses

**Health Literacy Index With 10 Score Increments**	**Regression Coefficient b (95% CI)^b^**		
	**Men (n =446)**	**Women (n =552)**	**Overall (n =998)**
Health status			
Self-perceived health status	0.034 (−0.13, 0.20)	0.34 (0.19, 0.48)***	0.21 (0.11, 0.32)***
Long-term illness	−0.12 (−0.18, −0.003)**	−0.05 (−0.009, 0.002)	−0.08 (−0.011, −0.002)**
Physical limitation related to health problem	−0.11 (−0.014, 0.001)*	−0.13 (0.014, −0.003)**	−0.12 (−0.012, −0.004)***
Health behaviors			
Smoking status	−0.12 (−0.016, −0.002)**	−0.16 (−0.016, −0.005)***	−0.13 (−0.014, −0.005)***
Doing exercise	0.11 (0.000, 0.017)**	0.71 (−0.004, 0.021)	0.12 (0.007, 0.025)**
Health care accessibility and utility			
Frequency of visiting doctors	−0.01 (−0.02, 0.03)	−0.05 (−0.07, −0.03)***	−0.03 (−0.04, −0.01)**
Accompanied to see doctors	0.02 (−0.05, 0.09)	−0.07 (−0.13, −0.001)*	−0.02 (−0.07, −0.03)

a Significant at *0.01<*P*<0.05; **0.001<*P*< 0.01; ***P<0.001. Health literacy index range from 0 to 50.

b Non-standardized regression coefficient adjusted for age, gender (for overall sample), marital status, education, social status, and ability to pay for medication.

## Discussion

The mean GHL in the study population was 34.0 ± 8.6 for men and 33.5 ± 9.4 for women, which was comparable with the studies from other countries (5, 18). The absence of association between gender and HL was comparable with the results from the European survey where gender had weak influence on the general health literacy (18).

Distribution of health literacy level in Kazakhstan was close to that in some European countries, e.g. Greece and Ireland (15). The proportion of respondents with inadequate and problematic health literacy was quite high (15.5% and 30.0%, respectively), yielding the proportion of respondents with limited HL equal to 45.5% (17). Different factors were found to be associated with health literacy (17–20). In the European survey, age was a predictor for health literacy with tendency for older groups to have lower health literacy (15, 17). In Dutch, adults with lower level of education, lower self-perceived social status, or male gender had lower health literacy (19). The health literacy study in Taiwan also identified negative association between age and health literacy(5). On the contrary, in this study, age was positively associated with health literacy exclusively in men. This could be explained in part by the society living experience, interactions with healthcare system, as well as knowledge related to health were heavily involved in health literacy. However, it is not clear similar factors did not play in women.

A positive association between age and health literacy regarding certain competencies, such as assessing health information, was found in some studies (19). Other studies demonstrated an increase of HL with age and a lower HL score among women compared with men (20). “Older age was also shown strongly associated with limited health literacy in reading comprehension, reasoning, and numeracy skills, while older age was weakly associated with limited health literacy in studies that measured health literacy as medical vocabulary” (2, 21). On the other hands, higher GHL was positively associated with socioeconomic and behavioral factors, such as ability to pay for medications, high self-assessed social status, moderate community involvement, watching health related TV programs. This was consistent with the results from other studies and underscores the importance of social and economic wellbeing for health literacy (5, 18). In European survey, self-assessed social status and education were found to be important predictors for health literacy, but not in this study that education was not statistically significant associated with GHL, as also observed in the study in Taiwan (5, 18). The results suggested, for instance, receiving university education did not necessarily add to one’s knowledge and skills related to health decisions and satisfactory interaction with healthcare system.

Health literate people had been shown with better health outcomes, whereas low health literacy was associated with chronic diseases (14, 22). A positive and significant association between self-perceived health status and health literacy, and negative associations between health literacy and health-related factors as long-term illness, limitation related to health, frequency of visiting doctors, and having somebody to accompany their visits to see the doctor. These findings highlighted the presence of the relationship between low health literacy and poor health outcomes and were consistent with other studies (5, 21, 23).

Health behavior was associated with health literacy (24, 25). Thus, interventions aimed to change risky behavior would improve health literacy (23). Further research should focus on the using the results of studying in the predictors of health literacy to work out approaches for behavior changes in the general public.

The study was with some strengths and limitations. Using internationally developed and validated instrument for the purpose of the study was a valuable asset that allowed making international comparisons. This study was the first attempt to our knowledge to examine and assess health literacy of Kazakhstani population. The limitation lied in the nature of a cross-sectional design.

## Conclusion

The level of general health literacy among residents of Almaty city was characterized as borderline sufficient. Almost half of the respondents had limited general health literacy, which indicated the need for activities to enhance health literacy. The associations revealed in the study demonstrated influence of demographic, socioeconomic and behavioral determinants on population’s health literacy. Further researches are needed to develop approaches to increase health literacy of the population to change health behavior and improve health outcomes.

## Ethical considerations

Ethical issues (Including plagiarism, informed consent, misconduct, data fabrication and/or falsification, double publication and/or submission, redundancy, etc.) have been completely observed by the authors.

## References

[1] WHO Health Impact Assessment: The determinants of health. Geneva 2015 http://www.who.int/hia/evidence/doh/en/

[2] Europe WHOROf The European health report 2005: Public health action for healthier children and populations: WHO Regional Office Europe; 2005 http://www.euro.who.int/__data/assets/pdf_file/0004/82435/E87325.pdf

[3] Sørensen K Van den Broucke S Brand H (2012). Health literacy and public health: a systematic review and integration of definitions and models. BMC Public Health, 12: 80. 22276600 10.1186/1471-2458-12-80 PMC3292515

[4] Kickbusch I Pelikan JM Apfel F Tsouros AD, editors. Health Literacy: The Solid Facts. WHO Regional Office for Europe. 2013 http://www.euro.who.int/__data/assets/pdf_file/0008/190655/e96854.pdf

[5] Duong VT Lin I-F Sørensen K (2015). Health Literacy in Taiwan: A Population-Based Study. Asia Pac J Public Health, 27(8): 871–80. 26419635 10.1177/1010539515607962

[6] Shreffler-Grant J Weinert C Nichols E (2014). Instrument to Measure Health Literacy About Complementary and Alternative Medicine. J Nurs Meas, 22(3): 489–99. 25608434 10.1891/1061-3749.22.3.489 PMC5185466

[7] Rosenbaum AJ Pauze D Pauze D (2015). Health Literacy in Patients Seeking Orthopaedic Care: Results of the Literacy in Musculoskeletal Problems (LIMP) Project. Iowa Orthop J, 35: 187–92. 26361464 PMC4492130

[8] Porter K Chen Y Estabrooks P (2016). Using Teach-Back to Understand Participant Behavioral Self-Monitoring Skills Across Health Literacy Level and Behavioral Condition. J Nutr Educ Behav,, 48(1): 20-6.e1. 26453368 10.1016/j.jneb.2015.08.012 PMC4715922

[9] Keim-Malpass J Letzkus L Kennedy C (2015). Parent/caregiver health literacy among children with special health care needs: a systematic review of the literature. BMC Pediatr, 15: 92. 26242306 10.1186/s12887-015-0412-x PMC4525748

[10] DeWalt DA Hink A (2009). Health literacy and child health outcomes: a systematic review of the literature. Pediatrics, 124 Suppl 3: S265–74. 19861480 10.1542/peds.2009-1162B

[11] Sanders LM Federico S Klass P Abrams MA Dreyer B (2009). Literacy and child health: a systematic review. Arch Pediatr Adolesc Med, 163(2): 131–40. 19188645 10.1001/archpediatrics.2008.539

[12] Sanders LM Shaw JS Guez G Baur C Rudd R (2009). Health literacy and child health promotion: implications for research, clinical care, and public policy. Pediatrics, 124 Suppl 3: S306–14. 19861485 10.1542/peds.2009-1162G

[13] Chang LC (2011). Health literacy, self-reported status and health promoting behaviours for adolescents in Taiwan. J Clin Nurs, 20(1–2): 190–6. 20629822 10.1111/j.1365-2702.2009.03181.x

[14] Liu Y-B Liu L Li Y-F Chen Y-L (2015). Relationship between Health Literacy, Health-Related Behaviors and Health Status: A Survey of Elderly Chinese. Int J Environ Res Public Health, 12(8): 9714–25. 26295246 10.3390/ijerph120809714 PMC4555308

[15] Sørensen K Pelikan JM Röthlin F (2015). Health literacy in Europe: comparative results of the European health literacy survey (HLSEU). Eur J Public Health, 25(6): 1053–8. 25843827 10.1093/eurpub/ckv043 PMC4668324

[16] Sørensen K Van den Broucke S Pelikan J (2013). Measuring health literacy in populations: illuminating the design and development process of the European Health Literacy Survey Questionnaire (HLS-EU-Q). BMC Public Health, 13: 948. 24112855 10.1186/1471-2458-13-948 PMC4016258

[17] HLS-EU Consortium Comparative report of health literacy in eight EU member states. The European Health Literacy Project 2009–2012. http://ec.europa.eu/chafea/documents/news/Comparative_report_on_health_literacy_in_eight_EU_member_states.pdf

[18] van der Heide I Rademakers J Schipper M (2013). Health literacy of Dutch adults: a cross sectional survey. BMC Public Health, 13: 179. 23445541 10.1186/1471-2458-13-179 PMC3599856

[19] Stanton R Scott D Happell B (2016). Low knowledge of physical health behaviours is associated with poor diet and chronic illness in adults. Aust J Prim Health, 22(3): 226–32. 25705916 10.1071/PY14132

[20] Tiller D Herzog B Kluttig A Haerting J (2015). Health literacy in an urban elderly East-German population–results from the population-based CARLA study. BMC Public Health, 15: 883. 26357978 10.1186/s12889-015-2210-7 PMC4566302

[21] Kobayashi LC Wardle J Wolf MS von Wagner C (2016). Aging and functional health literacy: a systematic review and meta-analysis. J Gerontol B Psychol Sci Soc Sci, 71(3): 445–57. 25504637 10.1093/geronb/gbu161 PMC4834761

[22] Fraser SDS Roderick PJ Casey M (2013). Prevalence and associations of limited health literacy in chronic kidney disease: a systematic review. Nephrol Dial Transplant, 28(1): 129–37. 23222414 10.1093/ndt/gfs371

[23] Taggart J Williams A Dennis S (2012). A systematic review of interventions in primary care to improve health literacy for chronic disease behavioral risk factors. BMC Fam Pract, 13: 49. 22656188 10.1186/1471-2296-13-49 PMC3444864

[24] Geboers B de Winter AF Luten KA (2014). The Association of Health Literacy with Physical Activity and Nutritional Behavior in Older Adults, and Its Social Cognitive Mediators. J Health Commun, 19 Suppl 2: 61–76. 25315584 10.1080/10810730.2014.934933

[25] Suka M Odajima T Okamoto M (2015). Relationship between health literacy, health information access, health behavior, and health status in Japanese people. Patient Educ Couns, 98(5): 660–8. 25739344 10.1016/j.pec.2015.02.013

